# Morphological and molecular characterization of developing vertebral fusions using a teleost model

**DOI:** 10.1186/1472-6793-10-13

**Published:** 2010-07-06

**Authors:** Elisabeth Ytteborg, Jacob Torgersen, Grete Baeverfjord, Harald Takle

**Affiliations:** 1 Nofima Marin AS, Norwegian University of Life Sciences, NO-1432 Ås, Norway; 2 Norwegian University of Life Sciences, NO-1432 Ås, Norway; 3 AVS Chile SA, Imperial 0655, Of. 3A, Puerto Varas, Chile

## Abstract

**Background:**

Spinal disorders are a major cause of disability for humans and an important health problem for intensively farmed animals. Experiments have shown that vertebral deformities present a complex but comparable etiology across species. However, the underlying molecular mechanisms involved in bone deformities are still far from understood. To further explicate the mechanisms involved, we have examined the fundamental aspects of bone metabolism and pathogenesis of vertebral fusions in Atlantic salmon (*Salmo salar*).

**Results:**

Experimentally, juvenile salmon were subjected to hyperthermic conditions where more than 28% developed fused vertebral bodies. To characterize the fusion process we analyzed an intermediate and a terminal stage of the pathology by using x-ray, histology, immunohistochemistry, real-time quantitative PCR and *in situ *hybridization. At early stage in the fusion process, disorganized and proliferating osteoblasts were prominent at the growth zones of the vertebral body endplates. PCNA positive cells further extended along the rims of fusing vertebral bodies. During the developing pathology, the marked border between the osteoblast growth zones and the chondrocytic areas connected to the arches became less distinct, as proliferating cells and chondrocytes blended through an intermediate zone. This cell proliferation appeared to be closely linked to fusion of opposing arch centra. During the fusion process a metaplastic shift appeared in the arch centra where cells in the intermediate zone between osteoblasts and chondrocytes co-expressed mixed signals of chondrogenic and osteogenic markers. A similar shift also occurred in the notochord where proliferating chordoblasts changed transcription profile from chondrogenic to also include osteogenic marker genes. In progressed fusions, arch centra and intervertebral space mineralized.

**Conclusion:**

Loss of cell integrity through cell proliferation and metaplastic shifts seem to be key events in the fusion process. The fusion process involves molecular regulation and cellular changes similar to those found in mammalian deformities, indicating that salmon is suitable for studying general bone development and to be a comparative model for spinal deformities.

## Background

The vertebral column is the defining character of vertebrates providing the organism with a unique ability of movement, form and function. Obviously, abnormalities to this organ can lead to severe and often painful pathological conditions. Spinal disorders are a major cause of disability for humans and an important health problem for intensively farmed animals. A number of animal models have been used to further explore the pathology and revealed that vertebral deformities present a complex but comparable cross species etiology [1,2]. Morphological changes like altered bone formation and cell density, thinning of osteoblasts along with increased cell proliferation and cell death are changes found in spinal deformities and intervertebral disc degeneration (IDD) in mammals [3,4]. Discs from patients with spinal deformities further have ectopic calcification of the vertebral endplates and sometimes in the disc itself [5]. Cells of the mammalian disc are derived directly from the phylogenetically conserved notochord [6]. Whereas only remnants of the notochord exists in the nucleus pulposus (NP) in humans by the age of 4, the notochord persist throughout all life stages in teleosts. Spinal disorders in teleosts like sea bass, sea bream, rainbow trout, halibut and salmon [7-12] have mostly been descriptive and few molecular studies have been carried out. However, in Atlantic salmon (*Salmo salar*) compression (platyspondyly) and/or vertebral fusion (ankylosis) accounts for 9 out of 20 recently described vertebral deformities [13]. Spinal fusions involves transformation of intervertebral notochord tissue into cartilage, shape alterations of vertebral body endplates, mineralization of the intervertebral cartilage and replacement of intervertebral cartilage by bone [14], pathological processes resembling those of IDD in mammals.

Skeletogenesis in salmon involves activity from the three main bone and cartilage cell types; chondrocytes, osteoblasts and osteoclasts. Bone formation further occurs via two basic mechanisms; compact bone of the amphicoel and trabeculae is formed directly through intramembranous ossification, whereas the cartilaginous template is replaced by bone in the arch centra through endochondral ossification. Bone formation is brought about by a complex set of highly regulated molecular pathways, involving extracellular matrix (ECM) constituents (e.g. collagens and osteocalcin), signaling molecules (e.g. hedgehogs and bmps) and transcription factors (reviewed [15-17]). Some of the key transcription factors in bone metabolism include runx2 and osterix [18], involved in the differentiation of mesenchymal stem cells (MSC) into osteoblasts that express bone matrix (col1a) and matrix mineralizing (osteocalcin and osteonectin) genes. Early chondrocyte differentiation is controlled by sox9, which regulates transcription of col2a [19], the major ECM component of cartilage. Further, before endochondral ossification may occur, mef2c assures that chondrocytes mature into col10a producing hypertrophic cells [20]. Both mineralized bone and cartilage is remodeled through the activity of osteoclasts. These multinucleated cells provide and acidic environment, express cathepsins and matrix metalloproteinases (mmps) and are tartrate acid phosphatase resistant (TRAP). Hence mineralized matrix may be broken down [21,22]. The skeletal pathways described in mammals are currently being understood in teleosts. In a recent study, we investigated 20 genes for their role in salmon spinal column skeletogenesis [23]. However, the genetic interactions of bone and cartilage development are currently becoming more entangled, as chondrocytes and osteoblasts are shown to intersect through the formation of chondroid bone. This process has been described through normal maturation, differentiation plasticity and trans-chondroid ossification [24-26]. Though, the molecular pathways involved are still far from understood.

During the last decade problems with spinal disorders in salmon have been increasingly in focus due to the importance of this species in the aquaculture industry. To further elucidate the mechanisms involved in the development of vertebral deformities, we analyzed an intermediate and terminal stage of the fusion process at a morphological level by using radiography and histology and gene transcriptional changes using quantitative PCR (qPCR) and *in situ *hybridization (*ISH*). We found that loss of cell integrity and ectopic bone formation characterizes the development of spinal fusions. During the fusion process a metaplastic shift appeared in the arch centra where cells in the intermediate zone between osteoblasts and chondrocytes co-expressed mixed signals of chondrogenic and osteogenic markers. A similar shift also occurred in the notochord where proliferating chordoblasts changed transcription profile from chondrogenic to also include osteogenic marker genes. We suggest that hyperthermic induced development of spinal fusions involve a metaplastic shift in cells from the chondrocytic lineage. With this work, we bring forward salmon to be an interesting organism to study development of spinal fusions.

## Results

**Figure 1:**
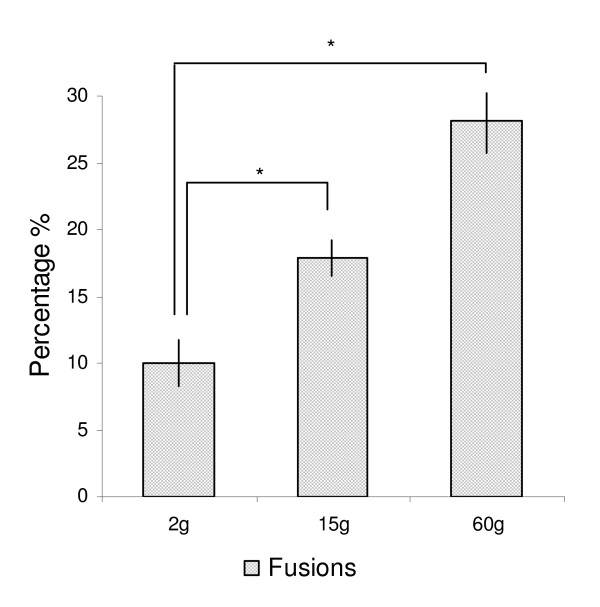
**Frequency of deformities**. Spinal fusions increased at each sampling point; 2 g, 15 g and 60 g. Each bar represents the means of analysis of variance at each sampling point as registered through radiographic findings in n = 4 tanks. Data are given in percentage ± st.dev, asterix indicate significant differences (P = 0.01).

**Figure 2:**
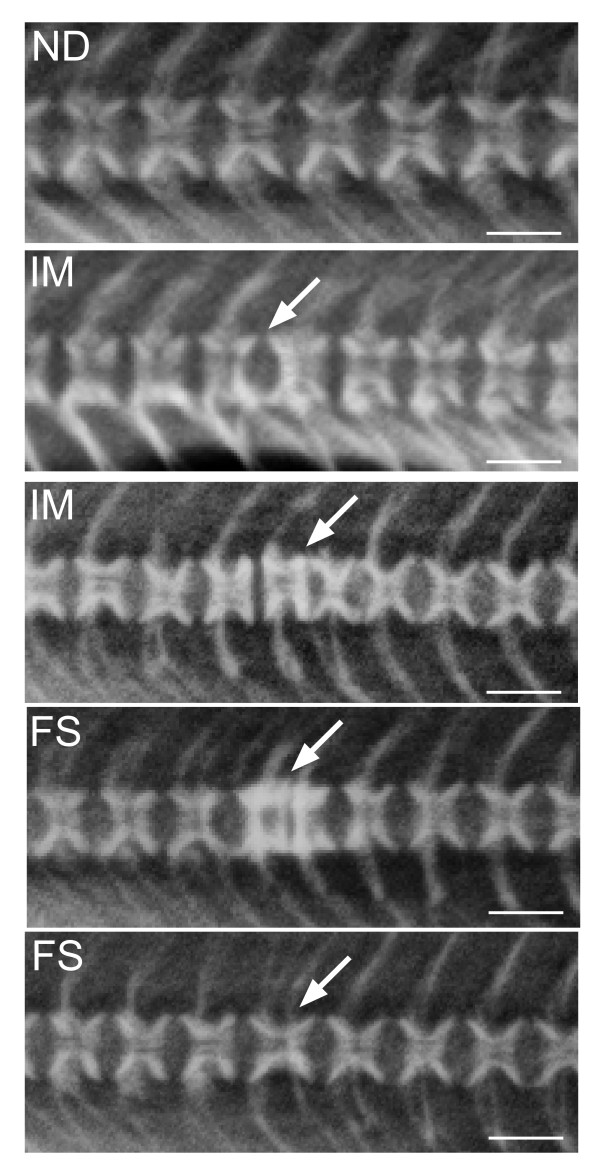
**X-ray and diagnostics**. The development of vertebral fusions is a dynamic process where the final result is a complete fusion of two or more vertebral bodies. Vertebrae were divided into non-deformed (ND), intermediate (IM) and fused (FS). White arrow points to malformed vertebral bodies. Scale bar = 0.1 cm.

The elevated temperature regime used in this study induced mainly vertebral deformities of the fusion type. The incidence of complete fusions was 10.0 (not significant = n.s.), 17.9 (p ≤ 0.001) and 28.1% (p ≤ 0.0001) at 2, 15 and 60 g, respectively (Figure 1). The incidence in the two later samplings are underestimated, since these numbers do not take into consideration that fish sampled at 2 and 15 g could develop into fusions at the following samplings. Some fish displayed more than one type of pathology, but pathological changes other than fusions were low in numbers and were not investigated. The fusion process is a dynamic process as visualized by x-ray in Figure 2.

### Histology and immunohistochemistry

**Figure 3:**
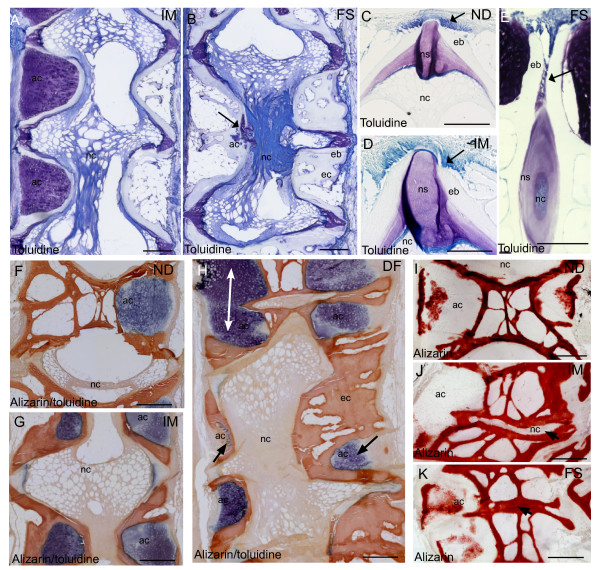
**Histological findings**. Toluidine blue staining of **A**. Intermediate vertebrae with irregular shaped vertebral bodies and **B**. Fused vertebrae with ectopic bone formation and denser notochord. Remnants of the arch center are present at ventral side of the notochord (arrow). Growth zones of vertebral endplates in **C**. Non-deformed **D**. Intermediate and **E**. Fused vertebrae. Osteoblasts appeared more disorganized throughout the pathology (arrow). At fused stage, osteoblasts located abaxial between vertebral bodies were observed. Double staining with Alizarin red S and toluidine blue of **F**. Non-deformed, **G**. Intermediate and **H**. Deformed vertebrae. Ectopic bone formation and reduced (arrow) or fused (white double arrow) arch centra was prominent throughout the developing pathology. Alizarin red S staining of **I**. Non-deformed **J**. Intermediate and **K**. Fused vertebrae. Notochordal tissue did not stain with alizarin red S until fusion was complete (arrow). ND, non-deformed; IM, intermediated; FS, fused, nc, notochord; ns, notochordal sheath, eb, endbone; ec, ectopic bone. Scale bar = 100 μm.

Histological examination revealed more detailed morphological characteristics of intermediate and fused vertebral bodies (Figure 3A, B). The osteoblasts at the growth zones of the vertebral endplate appeared well-organized in non-deformed vertebrae and little aberrancy was found when staining with toluidine blue (Figure 3C). The corresponding growth zones in intermediate vertebrae displayed alterations in vertebral endplates and more disorganized osteoblasts (Figure 3D). These findings became more pronounced at fused stage. The osteogenic zone of the vertebral endplate extended abaxial in-between two vertebral body endplates (Figure 3E). In addition, arch centra had decreased in fused vertebral bodies and chordocytes appeared denser compared to non-deformed (Figure 3B). Alizarin red S visualized more calcified tissue in areas with reduced arch centra in intermediate and fused vertebrae (Figure 3F-H). In fusions, normal vertebral hour-glass shape was replaced by a more compact and squared shape morphology, as the arch centra were more or less replaced by bone (Figure 3H). Alizarin red S stained calcified tissue and showed calcification of the centra and around hypertrophic chondrocytes (Figure 3I). No calcification was detected in the intervertebral space of incomplete fusions (Figure 3I). In fusions, growth zones of opposing vertebral bodies had fused and intervertebral space mineralized (Figure 3K).

**Figure 4:**
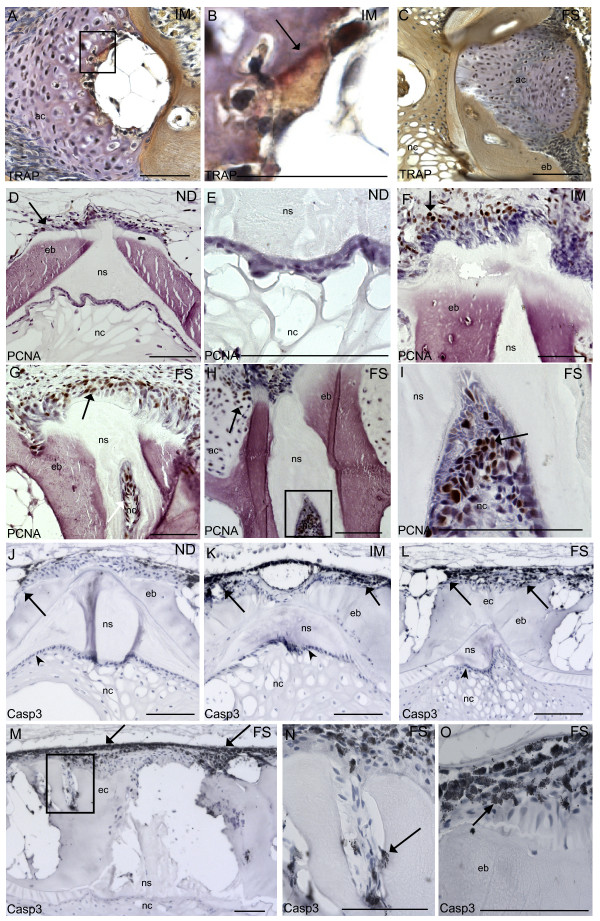
**Immunohistochemistry with TRAP, PCNA and Caspase 3**. **A**: One sample from intermediate group showed weak positive TRAP staining (arrow) at the ossifying border of the hypertrophic chondrocytes. **B**. Higher magnification of black box in A, positive TRAP staining (arrow). **C**. No TRAP activity was detected in any of the samples from the fused group. PCNA positive cells (brown) in **D**. Non-deformed. Some proliferating cells can be seen at the growth zones of the vertebral body endplate (arrow). **E**. Higher magnification of chordoblasts in non-deformed. Positive cells were rarely found in chordoblasts. **F**. Intermediate vertebrae. PCNA was detected in higher amount at growth zones of the vertebral body endplate and extending abaxial and axial direction (arrow). **G**. Fused vertebrae. PCNA labeled cells were detected in the corresponding areas, but in higher abundance (arrow). **H**. PCNA positive cells were observed in the notochord and also in arch centra (arrow). **I**. Higher magnification of the black box in H, PCNA positive notochordal cells (arrow). Caspase 3 positive cells (black) in **J**. Non-deformed. Caspase 3 positive cells can be seen at the fringe of the growth zones of the vertebral body endplate (arrow). No positive cells could be detected in the chordoblasts (arrowhead) in any of the groups. **K**. Intermediate vertebrae; positive cells were detected in exceedingly higher amount at the corresponding areas (arrows). **L**. Fused vertebrae; positive cells were detected in the corresponding areas, but in higher amounts (arrow). **M**. Positive caspase 3 signal increased along the rims of the vertebral body and in trabeculae in fused vertebral bodies where we observed ectopic bone formation (arrow). **N**. Higher magnification of black box in E showing caspase 3 positive cells in the joints of ectopic bone (arrow). **O**. Higher magnification of caspase 3 labeled cells showing typical apoptotic phenotype with membrane blebbing (arrow). ND, non-deformed; IM, intermediated; FS, fused, nc, notochord; ns, notochordal sheath, eb, endbone; ec, ectopic bone. Scale bar = 100 μm.

A balance between bone resorption and bone formation is required for maintaining bone integrity during remodeling. Thus, we examined osteoclast activity using TRAP staining. Weak positive TRAP staining was detected at the ossifying border of hypertrophic chondrocytes in the arch centra in one sample from the intermediate group (Figure 4A, B). No positive staining was found in samples from the fused group (Figure 4C).

To analyze if the morphological changes observed during development of fusions could be linked to an imbalanced cell cycling, we used immunohistochemistry with antibodies specific to PCNA for detection of proliferation and caspase 3 for detection of apoptosis. A few PCNA positive cells were apparent at the osteoblast growth zone at the endplates in non-deformed vertebral bodies (Figure 4D). PCNA positive cells were almost entirely restricted to these areas and were rarely found in chordoblasts or chordocytes (Figure 4E). However, we detected a markedly increase in PCNA positive cells at the growth zone of the endplates, and in cells extending axial at intermediate and fused stages (Figure 4F and 4G). Further, high abundance of proliferating chordoblasts were found in the notochord of vertebrae with reduced intervertebral space (Figure 4H and 4I). A few positive caspase 3 signals were detected at the rims of the osteoblast growth zone of the endplates in non-deformed vertebral bodies (Figure 4J). Increased caspase 3 signals were found in these areas of intermediate and fused vertebral bodies. Caspase 3 positive cells were also prominent at the transition between the intervertebral and vertebral regions (Figure 4K and 4L). The positive signal was further spreading along the rims of the vertebral bodies in axial direction (Figure 4M) and in cells harboring the joints of the trabeculae (Figure 4N). Caspase 3 was not detected in the notochord in any of the groups. The cells that stained positive had characteristic apoptotic morphology with membrane blebbing (Figure 4O).

### Spatial and temporal gene transcription in developing fusions

**Figure 5:**
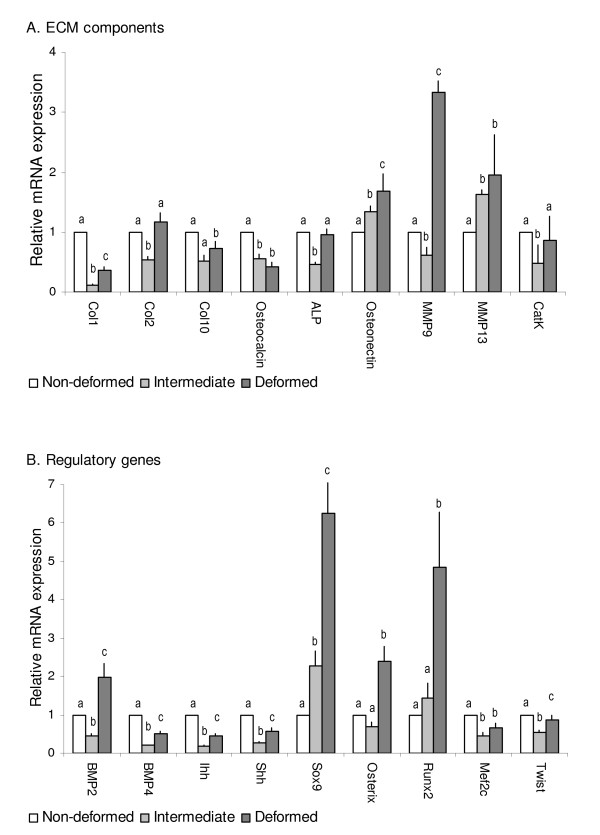
**Quantitative gene transcription profiles in intermediate and fused vertebral bodies**. Relative gene transcription of **A**. Extracellular matrix constituents and **B**. Regulatory genes in non-deformed (white bars) intermediate (light grey bars) and fused (dark grey bars) vertebrae, normalized with *ef1a*. Significant values (P = 0.05) indicated by a-b-c, n = 15, means ± SE. Transcription ratios are shown in relative mRNA expression along the y-axis, genes along the x-axis.

To examine transcriptional regulations involved in development of fusions, we analyzed non-deformed, intermediate and fused vertebrae with real-time qPCR, while the spatial gene transcription in intermediate and fused vertebrae were characterized by *ISH*. *ISH *of non-deformed vertebral bodies have previously been described in Ytteborg et al. [23]. No staining was detected for *ISH *with sense probes (additional file 1). Quantification of mRNA revealed that most genes were transcriptionally down-regulated during the pathogenesis of vertebral fusions and that the suppression was more profound at the intermediate stage than in fused specimens (Figure 5). We divided the 19 analyzed genes into two groups; structural genes and regulatory genes (transcription factors and signaling molecules).

### Structural genes

Nine out of 11 structural genes had a down-regulated transcription in the intermediate group compared to only five in the fused group (Figure 5A). Four genes were down-regulated in both groups, including genes involved in bone (*col1a1*) and hypertrophic cartilage ECM production (*col10a1*) and mineralization (*osteocalcin *and *alp*). *Col2a1 *transcription was down-regulated in intermediate while up-regulated in the fused group (n.s.). *Osteonectin *was up-regulated in both groups. Of genes involved in osteoclast activity, *mmp9 *showed opposite transcription, being down-regulated in intermediate while up-regulated in fused. *Mmp13 *and *cathepsin K *showed similar transcription pattern in the two groups, *mmp13 *up-regulated and c*athepsin K *down-regulated (n.s. in fused).

**Figure 6:**
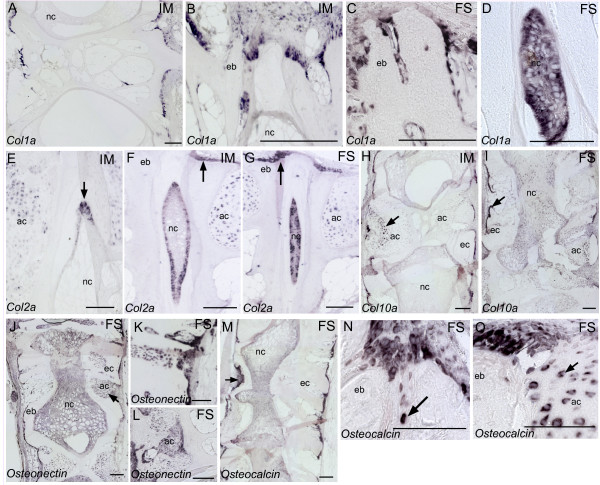
***In situ *hybridization of genes involved in the extracellular matrix**. **A**. *Col1a *transcription observed in trabeculae and at the growth zones of vertebral body endplates of intermediate vertebrae. **B**. *Col1a *in osteoblasts extending from the growth zones of the vertebral body endplate in intermediate and **C**. fused vertebrae. **D**. *Col1a *transcription in the notochord of a fusion. **E**. *Col2a *transcription in chordoblasts of intermediate vertebrae (arrow). **F**. *Col2a *transcription increased as intervertebral space narrowed down. In addition, *col2a *was observed at the osteoblast growth zone at the vertebral body endplates (arrow). **G**. In fused vertebrae, most cells in intervertebral space expressed *col2a *and expression in the osteoblasts increased (arrow). **H**. *Col10a *transcription along the rims of intermediate vertebrae and in hypertrophic chondrocytes (arrow). **I**. *Col10a *was more expressed in areas with ectopic bone formation (arrow). **J**. *Osteonectin *in fused vertebral bodies. Notice transcription along the rims of the vertebral bodies as well as in chondrocytes (arrow). Stronger staining was observed in areas with ectopic bone formation. **K**. *Osteonectin *transcription in areas with ectopic bone formation. **L**. *Osteonectin *transcribing chondrocytes. **M**. *Osteocalcin *was expressed in chondrocytes and along the rims of fused vertebrae. **N**. *Osteocalcin *transcription at the growth zone of two vertebral body endplates in a fusion. Notice cells expressing *osteocalcin *abaxial in-between the vertebral bodies (arrow). **O**. *Osteocalcin *expressing cells blending with chondrocytes. Both osteoblasts and chondrocytes expressed *osteocalcin *in these areas. ND, non-deformed; IM, intermediated; FS, fused, nc, notochord; ns, notochordal sheath, eb, endbone; ec, ectopic bone. Scale bar = 100 μm.

*ISH *analyzes of *col1a, col2a, col10a, osteonectin *and *osteocalcin *revealed cells exhibiting characteristics of both osteoblasts and chondrocytes. These findings were more pronounced in fused than intermediate specimens. *Col1a *was expressed in osteogenic cells along the rims of the vertebral body endplates and in osteoblasts at the lateral surfaces of trabeculae at the intermediate stage (Figure 6A). In incomplete fusions, we could locate osteogenic *col1a *positive cells in the growth zone of the vertebral endplate extending abaxial in-between vertebral bodies (Figure 6B, C). In addition, *col1a *was expressed in high abundance in the intervertebral space of incomplete fusions (Figure 6D). The chondrocytic marker *col2a *was observed in chordoblasts in intermediate samples (Figure 6E). Furthermore, *col2a *was expressed at the growth zone of the vertebral body endplates in both intermediate and fused samples (Figure 6F, G). Positive staining of *col2a *in the notochord became stronger as intervertebral space narrowed down (Figure 6F, G). Transcription of *col10a *was observed in hypertrophic chondrocytes and in osteogenic cells lining apical surfaces of trabeculae in intermediate and fused vertebrae (Figure 6H, I). *Col10a *seemed to be less expressed in both intermediate and fused vertebrae, as also observed from the down-regulated transcription of this gene from the qPCR results. *Osteonectin *showed a similar transcription pattern as *col10*, but transcription seemed increased in the trabeculae (Figure 6J). Transcription of *osteonectin *was also associated with chondrocytes in regions where arch centra fused (Figure 6K, L). Strong *osteonectin *transcription correlated with an up-regulated mRNA transcription observed from qPCR. *Osteocalcin *was transcribed in osteogenic cells lining surfaces of trabeculae of fused vertebrae (Figure 6M) and in cells located abaxial in-between two opposing vertebral body endplates (Figure 6N). When the vertebral growth zones blended with the arch centra, chondrocytes expressing *osteocalcin *was observed (Figure 6O).

### Regulatory genes - transcription factors and signaling molecules

All of the regulatory genes (Figure 5B) were less expressed in the intermediate compared to the fused group. Except of *osterix*, regulatory genes showed similar transcription patterns in the two groups. *Twist *involved in osteoblast inhibition and *mef2c *involved in chondrocyte hypertrophy were down-regulated in both groups. However, the chondrogenic marker s*ox9 *was up-regulated in both groups. The osteogenic markers *runx2 *and *osterix *had up-regulated transcription in the fused group, *runx2 *in intermediate group. Osterix was down-regulated in intermediate group, however n.s. Except of *bmp2 *in fused vertebral bodies, signaling molecules (*shh*, *ihh*, *pdgfrb *and *bmp4) *were down-regulated in both intermediate and fused group.

**Figure 7:**
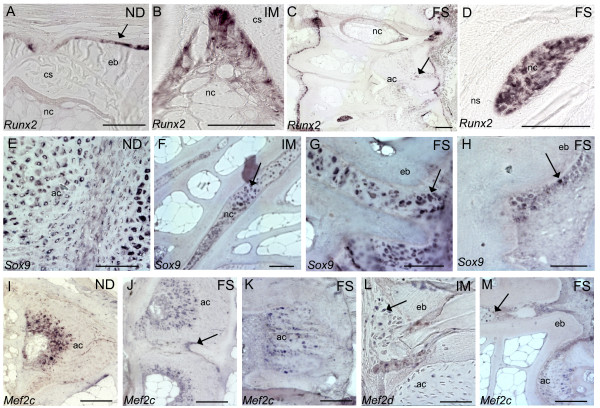
***In situ *hybridization of genes involved in regulatory processes**. **A**. *Runx2 *at the osteoblast growth zone of the vertebral body endplates in non-deformed vertebral bodies (arrow). Notice no transcription in chordocytes. **B**. *Runx2 *transcription in intermediate vertebrae showed transcription in chordoblasts. **C**. *Runx2 *transcription in fused vertebrae, notice positive staining in the chordocytes and in areas with ectopic bone formation (arrow). **D**. Strong staining of *runx2 *in cells in the notochord of a fusion. **E**. *Sox9 *transcription chondrocytes of non-deformed vertebrae. **F**. Fused vertebrae had high *sox9 *transcription in intervertebral space. **G**. Higher magnification of cells expressing *sox9 *at the vertebral growth zone of a fusion. **H**. *Sox9 *was also expressed along the rims of ectopic bone formation. **I**. *Mef2c *transcription in hypertrophic zone in non-deformed vertebral bodies. **J**. *Mef2c *in fusing arch centra. Notice positive cells outside the hypertrophic zone (arrow). **K**. *Mef2c *in arch center narrowing down. **L**. *Mef2c *transcription at the growth zones blending with the arch centra. **M**. *Mef2c *positive cells in intervertebral space of an incomplete fusion (arrow). ND, non-deformed; IM, intermediated; FS, fused, nc, notochord; ns, notochordal sheath, eb, endbone; ec, ectopic bone. Scale bar = 100 μm.

When analyzing selected genes by *ISH*, *runx2 *was never detected in chordocytes, chordoblasts or chondrocytes in non-deformed vertebral bodies. Positive *runx2 *staining was however detected at the osteoblast growth zone of the vertebral endplate (Figure 7A). In intermediate and fused samples we detected transcription at the corresponding growth zone and along the lateral surfaces of the trabeculae (Figure 7B). We observed an increased transcription of *runx2 *in the chordocytes of incomplete fusions (Figure 7C) and in the chordoblasts and chordocytes in more severe fusions (Figure 7D). These findings corresponded to the up-regulated transcription found by qPCR. *Sox9 *was expressed in chondrocytes in non-deformed vertebral bodies (Figure 7E) and in chordoblasts. In intermediate and fused samples, strong signals of *sox9 *were detected in intervertebral space (Figure 7F). *Sox9 *was also transcribed at the vertebral growth zones of the endplates (Figure 7G) and the signal was extending axial in severe fusions (Figure 7H). *Mef2c *was expressed in a wide zone of hypertrophic chondrocytes in non-deformed vertebral bodies (Figure 7I). Hypertrophic chondrocytes also transcribed *mef2c *in intermediate and fused vertebral bodies (Figure 7J). Further*, mef2c *was observed at the boundaries between two fused arch centra (Figure 7J). In fusions were arch centra narrowed down, *mef2c *transcription did not seem restricted to hypertrophic zones (Figure 7K). Some *mef2c *expressing cells was also detected at the vertebral endplates (Figure 7L) and abaxial between vertebral growth zones of opposing vertebral bodies in incomplete fusions (Figure 7M).

## Discussion

In this study we present a molecular characterization of mechanisms involved in development of vertebral fusions in salmon. We have previously shown that the non-deformed fish used in this study had indications of soft bone phenotype [23]. They were further characterized by disrupted chondrocytic maturation, increased zones of hypertrophic chondrocytes and delayed endochondral ossification in the arch centra [23]. The number of deformities increased throughout the experiment and an imbalanced bone and cartilage production characterized susceptible fish, predisposed for developing deformities [23]. In this study we wanted to analyze an intermediate and a terminal stage of the fusion process to further characterize developing deformities. Through this experiment, we found that vertebral deformities were developing through a series of events, of which five hallmarks were identified as particularly interesting. First, disorganized and proliferating osteoblasts were prominent in the growth zones of the vertebral body endplates. Second, a metaplastic shift made the borders less distinct between the osteoblastic growth zone and the chondrocytic areas in the arch centra. Third, the arch centra ossified and the endplates became straight, hence giving the vertebral bodies a squared shaped morphology. Fourth, the intervertebral space narrowed down and the notochord was replaced by bone forming cells. Fifth, in a complete fusion all intervertebral tissue was remodeled into bone.

One of the major morphological changes during the fusion process was ossification of the arch centra. Our findings suggest that this ectopic bone formation is a key event in development of vertebral fusions, which involve lack of normal cell differentiation and growth. Immunohistochemistry with PCNA showed that osteoblasts at the growth zone of the vertebral body endplates had a markedly increased cell proliferation during the fusion process. The increased proliferation of osteoblasts was apparently partly counteracted by increased cell death as shown by stronger caspase 3 signaling. Nevertheless, the osteoblasts at the vertebral endplates appeared less organized in intermediate and fused vertebral bodies by toluidine blue staining. In addition, in fused vertebral bodies we observed moderate changes of abaxial translocation of cells from the osteoblast growth zone. Abaxial direction of growth from the borders of vertebral body end-plates and formation of chondroid bone in these areas are also described in previous experiments [14,27]. The findings of increased proliferation and disorganized osteoblast growth were evident in vertebrae with modest alterations, which may suggest that this is an early event in the fusion process.

During the developing pathology, the marked border between the osteoblast growth zones and the chondrocytic areas connected to the arches became less distinct, as proliferating cells and chondrocytes blended through an intermediate zone. PCNA positive cells further extended along the rims of fusing vertebral bodies. This cell proliferation appeared to be closely linked to fusion of opposing arch centra. During the fusion process a metaplastic shift appeared in the arch centra where cells in the intermediate zone between osteoblasts and chondrocytes co-transcribed *col1a*, *col2a*, *runx2, osteocalcin *and *osteonectin*, as visualized by *ISH*. Based on histology, Witten et al. [25] have previously suggested the involvement of a metaplastic shift in developing fusions. In more progressed fusions, most cells in the arch centra seemed to co-transcribe osteogenic and chondrogenic markers. Our suggestion is therefore that trans-differentiated cells produce the ectopic bone.

Several *in vitro *studies have demonstrated that chondrocytes associated with calcifying cartilage can acquire properties of osteoblasts [28] and are able to change their phenotype from a primarily cartilage synthesizing cell type to a bone synthesizing cell type [29]. However, hypertrophic chondrocytes able to trans-differentiate into osteoblasts through a process called trans-chondroid ossification has also been described [24]. Interestingly, this type of growth has been identified during distraction osteogenesis in rats [24,30], a process where bone is formed rapidly upon stretching. During trans-chondroid ossification, chondrocytes are found to express both *col1 *and *col2 *[24]. In a review by Amir et al. [31] it was speculated if tension stress during distraction inhibited final differentiation of chondrocytes and rather trans-differentiated these cells into osteoblastic cells. At fused stage, early markers for osteoblasts and chondrocytes (*runx2, osterix, sox9 *and *bmp2) *were upregulated whereas the osteoblast inhibitor (*twist) *and genes involved in chondrocyte hypertrophy (*bmp4*, *mef2c, col10a, shh *and *ihh) *were downregulated, results also supported by *ISH*. Deletion of *Ihh *has been shown to disrupt the normal pattern of various zones of chondrocyte differentiation in the growth plate [32], whereas Sox9 accelerate chondrocyte differentiation in proliferating chondrocytes but inhibit hypertrophy [33]. Sustained *runx2 *expression, as found in our studies, is further associated with trans-differentiation of chondrocytes into bone cells [34]. On the contrary, analyzing the ECM components of both osteoblasts and chondrocytes (*col1a, col2a, col10a, osteocalcin *and *alp*) revealed that these transcripts had reduced activity in both intermediate and fused vertebrae. These findings might reflect the reduced radiodensity described in fish reared at elevated temperatures [23].

To further characterize the pathological bone formation in the chondrocytic areas in the arch centra, we analyzed osteoclast activity. Absence of osteoclasts visualized through TRAP staining was characteristic during the development of vertebral fusions, indicating that normal endochondral ossification was restrained. In addition, *cathepsin k *had a down-regulated transcription level. In normal developing salmon vertebrae, these areas are modeled through endochondral bone formation, a process requiring invasion of osteoclasts and activity of TRAP, Mmps and Cathepsin K [21,35]. Transcription of *mmp*s are up-regulated during IDD [36] and compression-induced IVD [37,38] in mammals. Intriguingly, *mmp9 *and *mmp13 *were also up-regulated during fusion of vertebral bodies in salmon. Excessive co-activity of *mmp9 *and *mmp13 *is linked to development and healing of chronic wounds in rainbow trout [39] and salmon [40]. Lack of osteoclast activity and reduced activity of genes involved in chondrocyte hypertrophy during development of vertebral fusions may therefore suggest that *mmp*'s were up-regulated in fused vertebral bodies as a response to chronic injury rather than bone resorption.

Our results suggest that the ossification type during development of spinal fusions and fast growth could be trans-chondroid ossification. A mixed type of intramembraneous and endochondral ossification, as suggested by Yasui et al. [24] and demonstrated by Okafuji et al. [30] may also occur, however the lack of osteoclast activity makes this less likely. Our findings indicate that chondrocytes had not only differentiated towards osteoblast-like cells, but also completed the differentiation to cells that were capable of producing mineralized bone matrix. Whether the suggested trans-chondroid ossification is trans-differentiation as a sudden switch from the chondrogenic to the osteogenic phenotype or a continuous differentiation was not assessed in this experiment. However, based on our results, a pathway to bone formation through chondrocytes might be possible during development of vertebral fusions.

The completing step in the fusion process is transformation of notochordal tissue into bone [14]. As intervertebral space narrowed down, proliferating chordoblasts and denser packet chordocytes were revealed through toluidine blue staining and PCNA antibody binding, respectively. The structured chordoblast layer increased and more of these cells stained for *col2a*. As the pathology progressed, proliferating chordoblasts seemed to occupy most of the intervertebral space and vacuolated chordocytes disappeared. Moreover, cells in the notochord had a transcription profile resembling the trans-differentiating cell at the borders between the osteoblast growth zones and the chondrocytic areas connected to the arches. Transcription of marker genes changed from chondrogenic to also include osteogenic, as mRNA of *osteocalcin*, *runx2, osteonectin *and *col1a *were detected. QPCR further showed up-regulated transcription of both *runx2 *and *sox9 *throughout the developing deformity. Comparative to our findings, disc cell proliferation and a switch in the synthesis of ECM components are associated with disc degeneration [41,42]. However, *ISH *revealed that whereas *sox9 *and *col2a *was present in chordoblasts from the non-deformed stage, *runx2 *and *col1a *was only detected in fused samples, when intervertebral space was severely narrowed. This co-transcription of chondrocytic and osteogenic markers in the notochord supports the hypothesis of a metaplastic shift during vertebral fusions in salmon [25].

The metaplastic shift in the notochord and arch centra may be induced to produce more robust cells, able to withstand increased mechanical load. However, as bone replaced chondrocytic areas throughout the pathology, notochordal tissue did not calcify until the deformity developed into severe fusion. We therefore suggest that metaplasia leads to cell types more suited to the new environment but that changes are related to a threshold of the stimuli, in this case, grade of fusion. A shift in NP cell population coincides with spinal disorders like IDD and changes in the synthesis of matrix molecules differ with the degree of degeneration [43]. A comparative pathological process to our findings is mammalian "Bamboo spine", describing a condition where vertebral bodies have fused and reshaped through ectopic bone formation [44,45]. Similar rescue processes have also been found in the mammalian AF, where it is strengthened through cartilage formation upon elevated mechanical load [46,47]. Overall, the vertebral fusion process seen in salmon might reflect an effort to restore and strengthen a vertebral area of a weakened vertebral column.

## Conclusion

Vertebral fusions develop through a series of events. Disorganized and proliferating osteoblasts at the growth zones and along the rims of affected vertebral bodies characterized the fusion process. Moreover, loss of cell integrity through cell proliferation was prominent at the border between the osteoblastic growth zone and the chondrocytic areas in the arch centra and in intervertebral space. During the fusion process a metaplastic shift appeared in the arch centra where cells in the intermediate zone between osteoblasts and chondrocytes co-expressed mixed signals of chondrogenic and osteogenic markers. A similar shift also occurred in the notochord where proliferating chordoblasts changed transcription profile from chondrogenic to also include osteogenic marker genes. As the pathology progressed, ectopic bone formation was detected in these areas. Since transcription turned from chondrogenic to osteogenic, our suggestion is that trans-differentiated cells produce the ectopic bone. In complete fusions, all intervertebral tissue was remodeled into bone. The molecular regulation and cellular changes found in salmon vertebral fusions are similar to those found in mammalian deformities, showing that salmon is suitable for studying general bone development and to be a comparative model for spinal deformities. With this work, we bring forward salmon to be an interesting organism to study general pathology of spinal deformities.

## Methods

### Rearing conditions

This trial was performed under the supervision and approval of the veterinarian that has appointed responsibility to approve all fish experiments at the research station in accordance to regulations from the Norwegian authorities regarding the use of animals for research purposes. The experiment was carried out at Nofima Marins research station at Sunndalsøra, Norway, in 2007, as described in Ytteborg et al. [23]. During egg rearing, water supply was continuous from temperature controlled tanks stabilized at 10 ± 0.3°C. The temperature was gradually increased at first feeding to 16 ± 0.3°C (1°C per day). Temperatures exceeding 8°C during egg rearing and 12°C after start feeding elevate the risk of developing spinal fusions.

### Radiography and classification

Sampling was directed from radiographs so that the sampled area corresponded to the deformed or normal area. Fish were sedated (Tricaine methane sulfonate, Pharmaq, Norway) and radiographed during the experiment at 2 g, 15 g and 60 g. Fish that were not sampled were put back into oxygenated water to ensure rapid wakening. The x-ray system used was an IMS Giotto mammography system (model number 6020/3, IMS Giotto, Bologna, Italy) equipped with a FCR Profect image plate reader and FCR Console (Fuji Medical Inc., Japan). At 15 g size, fish were sampled for histological and gene transcriptional analysis. Samples for *ISH *and histology were fixed in 4% PFA (n = 24) and samples for RNA isolation were snap frozen in liquid nitrogen and stored at -80°C (n = 45).

All fish were divided into three categories where the first group was non-deformed. These spinal columns had no observable morphological changes in the vertebral bodies or in intervertebral space. We further sampled vertebral areas at two different stages in the pathological development of fusions, termed intermediate and fused. Vertebrae diagnosed as intermediate included various degrees of reduced intervertebral space and compressions. Samples characterized as fused ranged from incomplete fusions to complete fusions.

### Statistical analyses

Incidence of fusions were observed through radiography and calculated using a one-way analysis of variance model (GLM procedure, SAS 9.1 software, SAS Institute Inc., USA). Results are represented as means ± standard deviation (st.dev).Statistics for mRNA transcription analysis are described in the real-time PCR chapter.

### Sample preparation

Histological staining and *ISH *was carried out on five μm Technovit 9100 ^®^New sections according to the protocol [48]. Serial sections were prepared in the parasagittal orientation from vertebral columns, starting at the periphery and ending in the middle plane of the vertebrae using a Microm HM 355S (Thermo Fisher Scientific Inc., MA, USA). For immunohistochemistry, tissue was decalcified for seven days in 10% EDTA, dehydrated in ethanol, cleared and embedded in paraffin. Five μm serial sections were prepared as described above, de-waxed with Clear Rite (Richard-Allan, MI, USA), followed by two times washing in xylene (Merck Chemicals Ltd.) for five min each. Sections were then rehydrated before rinsed in dH_2_O.

### Histology and immunohistochemistry

Bone and cartilage formation in the spinal columns were assayed by Alizarin Red S/Toluidine Blue (Sigma-Aldrich, MO, USA) staining. Sections were stained for 5 min in Alizarin red (pH 4.2) and for 2 min in 0.1% Toluidine blue (pH 2.3), with a brief rinse in dH _2_O in between. Single staining with the two dyes was also performed. All sections were dehydrated in ethanol and mounted with Cytoseal 60 (Electron Microscopy Science, PA, U.S.A) prior to microscopy. To demonstrate osteoclast activity, TRAP was visualized with the Acid phosphatase leukocyte kit No. 387 (Sigma-Aldrich) was applied according to the manufacturer's protocol, with the exception of a 2 h incubation at 37°C. Subsequently, slides were rinsed in dH_2_O and counterstained with Mayers hematoxylin (Sigma-Aldrich) for 30 s. Cell proliferation and apoptosis were assessed by immunohistochemical detection of proliferating cell nuclear antigen (PCNA) and cleaved Caspase 3, respectively [49-51]. Slides were placed in 0.1 M citric acid, 0.05% Tween 20 (pH 6) and heated in microwave, 5 min at 900 W and 4 min at 650 W. Endogenous peroxidase activity was blocked 10 min in 3% H_2_O_2 _in methanol. The sections were washed 3× in PBS and incubated with a mouse anti PCNA monoclonal antibody (clone PC10, Zymed Laboratories Inc., CA, USA) or Cleaved Caspase 3 (Asp175 5A1, Cell Signaling Inc. Boston, MA, USA), following the manufacturer's instructions. Slides were washed 3× 5 min in PBS-Tween 20 before counterstained with Mayer's hematoxylin for 2 min, washed in water, dehydrated in a graded series of ethanol solutions, cleared with xylene, and mounted with Cytoseal60 (Electron Microscopy Science). Controls were incubated without substrate. Microscopic analyses were performed by the stereomicroscope Zeiss Axio Observer Z1 using brightfield illumination and digitized images obtained with an AxioCam MRc5 camera using AxioVision software (Carl Zeiss Microimaging GmbH, Göttingen, Germany).

### Primer design

Primers (Table 1) for transcription analysis were based on known salmon sequences or on conserved regions of known teleost sequences paralogues. Primers were designed using the Vector NTI Advance 10 (Invitrogen, CA, USA) and NetPrimer (PREMIER Biosoft, CA, USA) software. All PCR products were cloned using pGEM T-easy (Promega, WI, USA) and sequenced with Big Dye Terminator chemistry and the ABI 3730 automated sequencer, both delivered by (Applied Biosystems, CA, USA). The obtained salmon clones were analyzed by BLAST and deposited in the Genbank database.

**Table 1:** Primers used for cloning, sequencing, transcriptional analysis and probe synthesis

Gene	Orientation	Genbank	Use	Sequence (5'-3')
Extracellular Matrix constituents				

Col1a1	Forward	FJ195608	RT	AGAGAGGAGTCATGGGACCCGT
	Reverse		RT	GGGTCCTGGAAGTCCCTGGAAT
	Forward		ISH	TAGCCGTGGTTTCCCTGGTT
	Reverse		ISH	CCGGGAGGTCCAAATCTACC
Col2a1	Forward	FJ195613	RT	TGGTCGTTCTGGAGAGACT
	Reverse		RT	CCTCATGTACCTCAAGGGAT
	Forward		ISH	GCTGGCGAGACAGGAGAGA
	Reverse		ISH	GCCTCATCAGCCCTCATGTA
Col10a1	Forward	EG837148	RT	TGGTGCTCTTTGACTGCCTGTAA
	Reverse		RT	CATCCTGTGTGTTGCAATATCACA
	Forward		ISH	AACAAGGGCTTCTTGGATCA
	Reverse		ISH	CATAATGCATCCTCAGGCAT
Alp	Forward	FJ195609	RT	CTAGTTTGGGTCGTGGTATGT
	Reverse		RT	TGAGGGCATTCTTCAAAGTA
Osteocalcin	Forward	FJ195616	RT	GTGAACCAACAGCAAAGAGA
	Reverse		RT	CCAGGTCCTTCTTAACAAACA
	Forward		ISH	CTCATACTTGTTGATCGTCCAG
	Reverse		ISH	TCTTTCTCTCTCGCTCTCCC
Osteonectin	Forward	FJ195614	RT	ATTACTGAGGAGGAGCCCATCATT
	Reverse		RT	CCTCATCCACCTCACACACCTT
	Forward		ISH	CTGAACGATGAGGGTGTGGA
	Reverse		ISH	CGAGTGGTGCAGTGCTCCAT
Mmp9	Forward	CA342769	RT	AGTCTACGGTAGCAGCAATGAAGGC
	Reverse		RT	CGTCAAAGGTCTGGTAGGAGCGTAT
Mmp13	Forward	DW539943	RT	TGATGTCCAAGTCAGCCGCTTC
	Reverse		RT	TGGTCTGCCACTTGCGATTGTC
Cathepsin K	Forward	NM_001140399	RT	ATGACCAACGCCTTCGAGTAC
	Reverse		RT	AAGGTGGAGAGGGTGGCATC

Transcription factors				

Sox9	Forward	EU344852	RT	CCTGCAAACAAGACAAGGT
	Reverse		RT	GGGTCGAGTAGATTCATACGA
	Forward		ISH	GGGGATACTATTTGACTGGATC
	Reverse		ISH	TCTGTCTTGATGTGTGTGGG
Runx2	Forward	FJ195615	RT/ISH	CCACCAGGGACAGACACAGAT
	Reverse		RT/ISH	GAACGGACTGAGATCTGACGAA
Osterix	Forward	FJ195612	RT	TCCCATAGACTTTCCCACA
	Reverse		RT	TGCCTCAGGACATGTACAA
Mef2c	Forward	GU252207	RT	CACCGTAACTCGCCTGGTCT
	Reverse		RT	GCTTGCGGTTGCTGTTCATA
	Forward		ISH	GACAGAGACTGTGTGGTGTTCCCT
	Reverse		ISH	AGGTGGAGGGAGCTACCACTGTTA

Signalling molecules				

Bmp4	Forward	FJ195610	RT	TCAAGTTGCCCATAGTCAGT
	Reverse		RT	CACCTGAACTCTACCAACCA
Bmp2	Forward	BT059611	RT	ATGTGGTATTGCACCCATT
	Reverse		RT	ATGGACAGTTTCCCAATGA
Shh*	Forward	AY370830	RT	CCGGCTCATGACTCAGAGATG
	Reverse		RT	TATCCCTGGCCACTGGTTCA
Ihh	Forward	FJ195617	RT	CAGATGACCCACTGGACTGAT
	Reverse		RT	GCTTGGTTGGGAGATATGCA

Housekeeping gene				

Ef1a **	Forward	DQ834870	RT	CACCACCGGCCATCTGATCTACAA
	Reverse		RT	TCAGCAGCCTCCTTCTCGAACTTC

*Wargelius et al. [54] **Olsvik et al [52]

### RNA isolation and cDNA synthesis

Tissue homogenization from 15 replicates from each group was achieved in a mortar with liquid nitrogen. RNA was extracted using Trizol reagent and Micro to Midi Kit^® ^(Invitrogen). Brief, tissue was homogenized in a mortar with liquid nitrogen and total RNA was extracted using Trizol reagent and Micro to Midi Kit^® ^(Invitrogen) before DNase treatment (DNase1, Invitrogen). The quality of the RNA was assessed spectrophotometrically (NanoDrop Technologies, DE, USA) 1 μg RNA was reverse transcribed to cDNA using oligo(dT) primer and the Taqman Gold RT-PCR kit (Applied Biosystems). The cDNA synthesis was performed with 10 min primer incubation at 25°C, 1 h RT step at 48°C and 5 min RT inactivation at 95°C. All reactions were performed in accordance to the manufacturer's protocol.

### Real-time quantitative RT-PCR

Real-time qPCR was conducted using the Light cycler 480 and SYBR Green chemistry (Roche, Switzerland) at the following thermal cycling conditions: 95°C for 10 min, followed by 45 cycles at 95°C for 15 s, 60 ± 1°C for 15 s and 72°C for 15 s. Further, specificity was assessed by the melting curves, determined post PCR (95°C for 15 s, 60 ± °C for 1 min and 97°C continuous). To determine the efficiency of target genes and reference gene (*ef1a*), we used the standard curve method. Relative target gene mRNA was normalized to relative *ef1a *mRNA levels for all sample, as recommended by Olsvik et al. [52]. The transcription ratios were analyzed using the Relative Expression Software Tool (REST) and tested for significance by the Pair Wise Fixed Reallocation Randomization Test^© ^[53].

### In situ hybridization

Digoxigenin labeled antisense and sense riboprobes were synthesized according to the manufacturer's protocol (Roche), using 250 ng of SP6 and T7 tailed PCR fragments as template. *ISH *was carried out on five μm Tw9100 sections as described [48], and microscopic analyses of the NBT/BCIP stained sections were conducted on a Zeiss Axio Observer Z1 equipped with an AxioCam MRc5 camera and AxioVision software (Carl Zeiss Microimaging GmbH).

## Abbreviations

Alp: alkaline phosphatase; BCIP/NBT: 5-bromo-4-chloro-3 indolyl phosphate *p*-toluidine salt/nitro blue tetrazolium chloride; Bmp: Bone morphogenetic proteins; Col1a1: collagen type 1a1; Col2a1: collagen type 2a1; Col10a1: collagen type 10a1; Ef1a: Elongation factor 1; Ihh: Indian hedge hog; IVD: intervertebral disk degeneration; IDD: Intervertebral disc disease; ISH: In situ hybridization; Mef2c: myocyte enhancer factor 2c; Mmp: Matrix metalloproteinase; PBS: phosphate-buffered saline; PFA: paraformaldehyde; qPCR: quantitative polymerase chain reaction; Runx2: runt-related transcription factor 2; Shh: Sonic hedge hog; Sox9: (sex determining region Y) box 9; TRAP: Tartrate resistant acid phosphatase.

## Authors' contributions

EY participated in sampling, carried out the molecular studies, microscopic analyzes and acquisition and interpretation of data and drafted the manuscript. JT participated in the design of the *ISH *experiments, sequence alignment and probe design, microscopic analyzes and acquisition and interpretation of data. GB carried out the radiological diagnostics, accompanied in statistical analysis and participated in the experimental design. HT conceived the study and its experimental and molecular design, coordinated the sampling, and participated in acquisition and interpretation of data and in the drafting of the manuscript. All authors read and approved the final manuscript.

## Supplementary Material

Additional file 1 **Sense probes**. No staining was detected for *ISH *with sense probes. nc, notochord; ns, notochordal sheath, eb, endbone; tb, trabecular bone. Scale bar = 100 μm. Click here for file
