# Supplementary figures and images for: Morphological and molecular characterization of developing vertebral fusions using a teleost model
Source: BMC Physiol. 2010 Jul 6;10:13. doi: 10.1186/1472-6793-10-13 (PMC2909226; doi:10.1186/1472-6793-10-13)

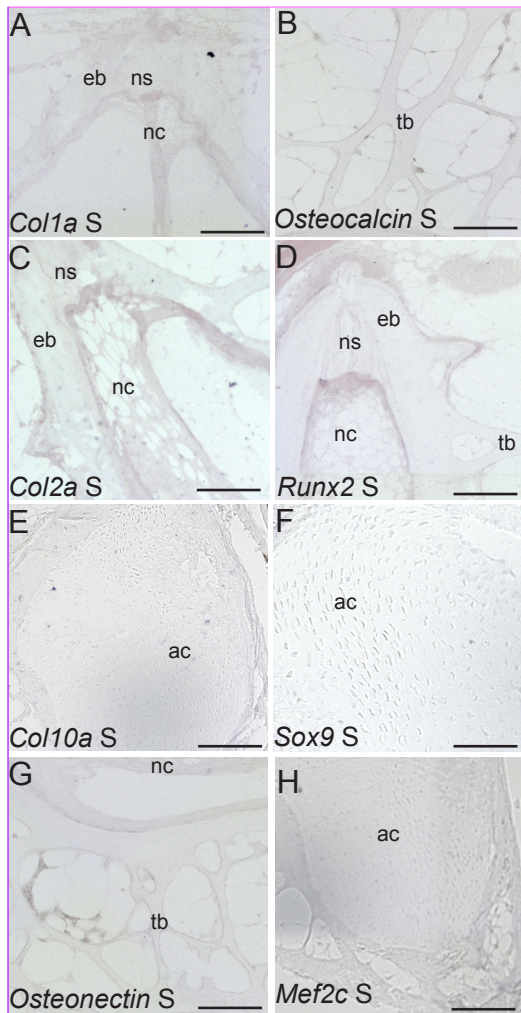

Supplement: Additional file 1 — Sense probes. No staining was detected for ISH with sense probes. nc, notochord; ns, notochordal sheath, eb, endbone; tb, trabecular bone. Scale bar = 100 μm. [file 1472-6793-10-13-S1.PDF]
